# Vibrational Mode-Specific Dynamics of the OH + XO→ H + XO_2_ (X = C/S) Reactions: Similar Topologies, Different Dynamics

**DOI:** 10.3390/molecules31173041

**Published:** 2026-08-29

**Authors:** Jie Qin, Weihao Zeng, Penghui Li, Ting Long, Jun Li

**Affiliations:** 1 School of Chemistry and Chemical Engineering & Basalt Fiber and Composite Key Laboratory of Sichuan Province, Sichuan University of Arts and Science, Dazhou 635000, China; 2 School of Chemistry and Chemical Engineering & Chongqing Key Laboratory of Chemical Theory and Mechanism, Chongqing University, Chongqing 401331, China

**Keywords:** quasi-classical trajectory, vibrational mode-specific, reaction dynamics

## Abstract

The prototypical complex-forming reactions of OH + CO and OH + SO are critical elementary processes in atmospheric chemistry and combustion systems. The vibrational mode-specific quasi-classical trajectory (QCT) investigations for these two reactions were performed on full-dimensional globally accurate potential energy surfaces (PESs). Integral cross-sections (ICS), differential cross-sections (DCS), and product energy distributions for different vibrational excitation modes were calculated and analyzed. Although both exothermic oxygen-transfer reactions share similar topological features in their PES profiles, their energy landscapes exhibit pronounced differences, giving rise to entirely distinct dynamic and kinetic behaviors. This work will advance our understanding of how PES topology, collision energy, and mode-specific vibrational excitation jointly govern reactivity and energy dissipation in radical oxidation processes involving carbon- and sulfur-containing oxides.

## 1. Introduction

Hydroxyl radical (OH)-initiated oxidation of small inorganic oxides (CO, SO) represents a class of core elementary reactions governing atmospheric chemistry [1,2] and fossil fuel combustion processes [3]. The radicals play irreplaceable functions in tropospheric ozone cycling [4,5], acid rain formation [6,7], flame radical pool balance [8], and pollutant transformation mechanisms [9,10,11,12]. These two prototypical exothermic oxygen-transfer reactions, OH + CO → H + CO_2_ [13,14,15,16,17,18] and OH + SO → H + SO_2_ [19,20,21,22,23,24,25], share similar overall topological frameworks. However, distinct differences in their potential energy surface (PES) topologies, intermediate complex well depths, and the presence or absence of entrance-channel energy barriers give rise to fundamentally different kinetic and dynamic behaviors. The properties render them ideal comparative model systems for exploring the intrinsic mechanisms of mode-specific dynamics in radical oxidation processes.

Extensive theoretical and experimental studies have been carried out on the OH + CO reaction system [13,26,27,28,29,30,31,32,33,34]. Early crossed molecular beam studies revealed a forward–backward asymmetric differential cross-section, indicative of a long-lived HOCO intermediate [29,34,35,36,37,38]. Kudla and Schatz [39] found that vibrational excitation of C–O and O–H bonds leads to modest increases in the cross-section, with the effect being larger for OH excitation than for CO excitation. They supposed that vibrational excitation does not produce mode-selective enhancements, which is dominant at low energies. Spencer et al. [40] detected the vibrational excitation by electron paramagnetic resonance and reported that vibrational excitation of OH enhances the reaction with CO by about a factor of 2. Numerous theoretical and experimental investigations have focused on the effects of rovibrational excitation on the reaction rate constants [41,42,43,44,45,46], while fewer studies have been devoted to reaction cross-sections and product energy distributions [34,39]. The role of quantum tunneling is confirmed to be extremely important at the lower temperatures, which supports by recent work regarding the loss of hydrogen atoms steps, such as through TS4 [14,38,39,47,48,49,50,51,52,53]. Theoretically, the reaction features a challenging PES topography with deep potential wells and a near-isoenergetic exit barrier. Although recent permutation-invariant polynomial–neural network (PIP-NN) PESs [2,54] have significantly improved the accuracy of dynamical predictions, the calculated differential cross-section (DCSs) on that PES did not agree with the experimental results. A combined experimental-theoretical study was performed to advance the understanding of the dynamics of this reaction; good agreement was found between experiment and theory, both for product angular and translational energy distributions [14].

The quasi-classical trajectory (QCT) method has been extensively applied to study the kinetics and dynamics of the OH + SO reaction, based on the previously developed double many-body expansion (DMBE) potential energy surface by Ballester et al. [22,23,25,55]. Then, they studied state-specific cross-sections and thermal rate coefficients for OH (*v* = 1,5) vibrational deactivation by SO collisions, revealing selective energy transfer pathways [56]. The integral cross-section (ICS) and product energy partitioning of the entire reaction have yet to be thoroughly studied.

**Figure 1:**
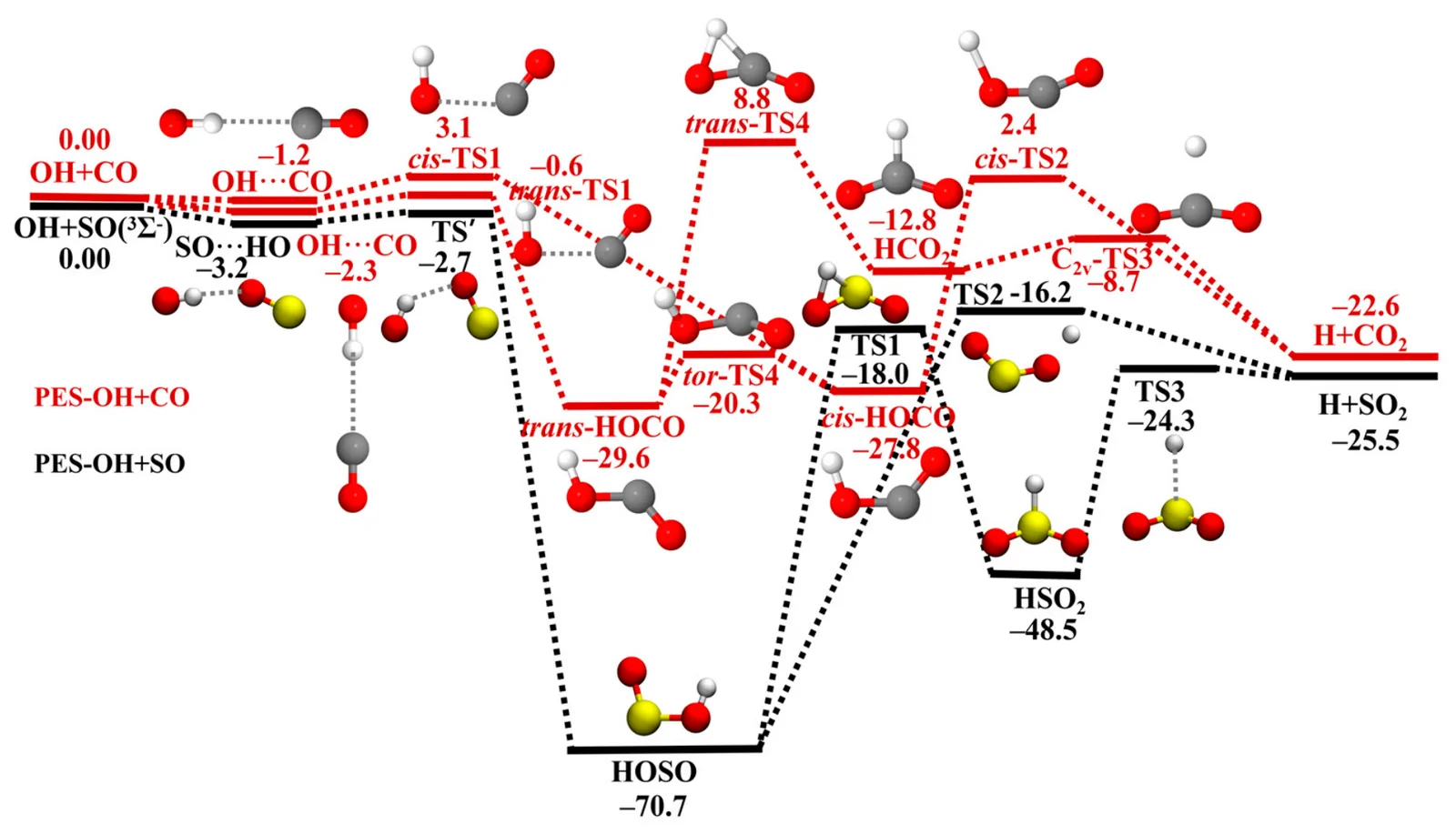
Schematic reaction profile of the OH + CO [14,16] (red) and OH + SO (black) [19] reactions. All energies (in kcal mol^−1^) are relative to the respective reactant asymptote on the PES.

Figure 1 presents the one-dimensional potential energy profiles of the OH + CO (red line) and OH + SO (black line) reactions on their PIP-NN PESs [14,16,19], PES-OH + CO and PES-OH + SO. The two PESs share similar topological evolution, both following the same elementary-step progression, as follows: reactants → first transition state → first intermediate → second transition state → second intermediate → third transition state → products. Thermodynamically, both reactions are exothermic, with product energies lying below those of the reactants, rendering both processes spontaneous. Mechanistically, each reaction proceeds via oxygen transfer from the hydroxyl radical to the small-molecule substrate, yielding a hydrogen atom and the corresponding stable-oxide product, and their reaction frameworks are highly consistent.

However, marked differences exist between the two systems. The OH + CO reaction features a prominent energy barrier height, reaching 8.8 kcal mol^−1^ [14] above the reactant asymptote, which serves as the rate-determining step. Consequently, a substantial energy threshold must be overcome for the reaction to proceed at ambient temperatures. By comparison, all transition states and intermediates in the OH + SO reaction lie below the reactant energy, with no positive forward barrier. The OH + SO system forms a deep intermediate with an energy as low as −70.7 kcal mol^−1^, conferring exceptional stability, whereas the most stable intermediate in the OH + CO pathway reaches only −29.6 kcal mol^−1^. Thus, although the two reactions share similar elementary reaction channels, their potential energy contours are fundamentally different. The OH + SO reaction is barrierless, possesses a more stable intermediate, and releases greater heat. In contrast, the reactivity of the OH + CO reaction remains constrained by the rate-determining barrier situated above the reactant asymptote.

In this work, we present a comparative quasi-classical trajectory study on chemically accurate full-dimensional PIP-NN PESs [16,19] for OH + CO → H + CO_2_ and OH + SO → H + SO_2_ to investigate the vibrational mode-specific effect—namely, the influence of the selective excitation of the OH or SO/CO vibrational modes on the reactivity and dynamics. We quantitatively compare the two reactions across four key dimensions, as follows: collision energy dependence of ICSs, mode-specific promotion/inhibition effects, differential cross-sections, and final product energy distributions.

## 2. Results and Discussion

### 2.1. OH + CO

**Figure 2:**
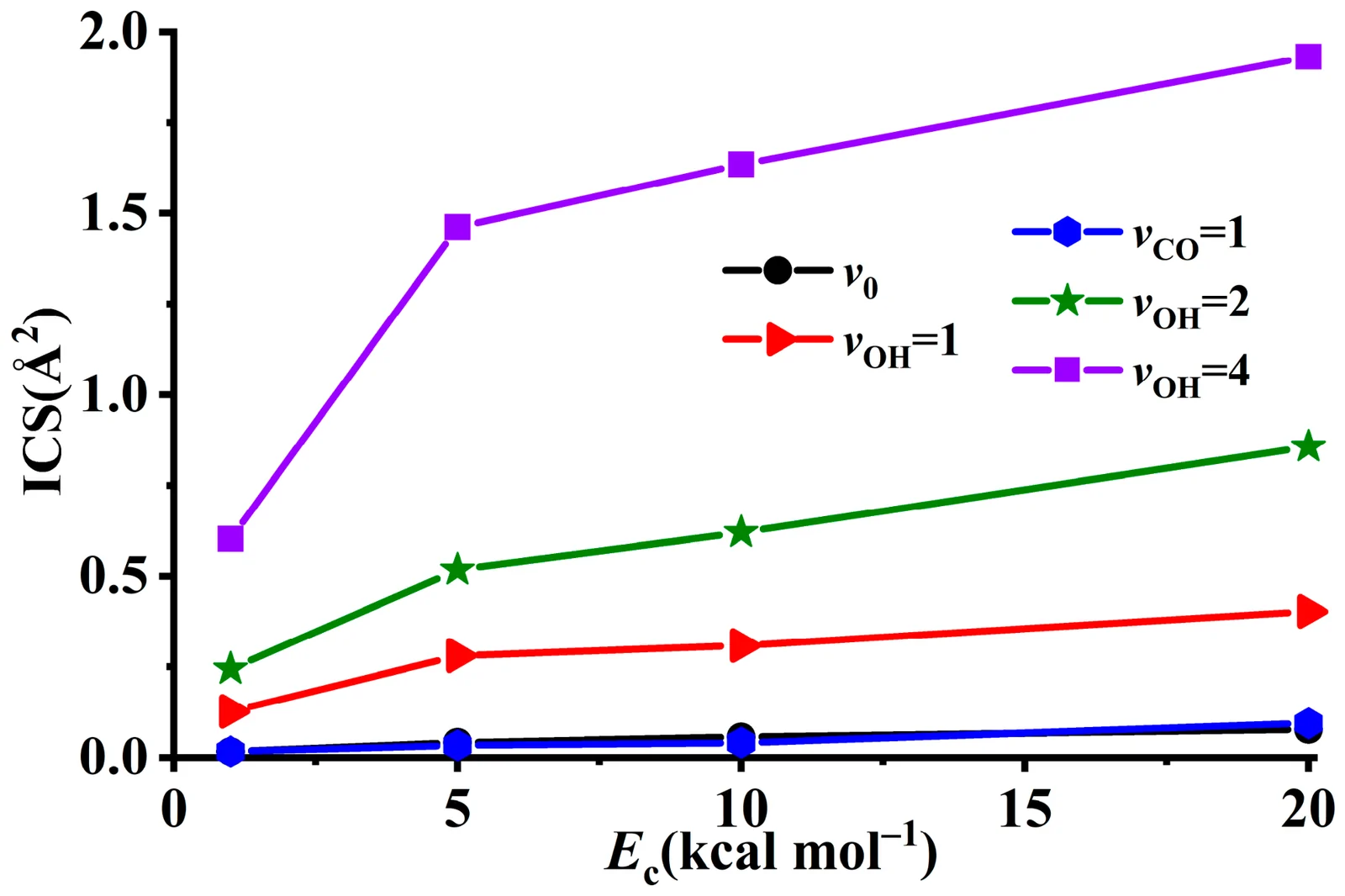
The calculated ICSs for the reaction OH + CO using the PES-OH + CO (this work) [16] are plotted as a function of collision energy.

The ICSs for vibrational states (*v*_0_, *v*_OH_ = 1, *v*_CO_ = 1, *v*_OH_ = 2, and *v*_OH_ = 4) of the OH + CO reaction are shown in Figure 2. Clearly, the ICSs of the OH + CO reaction increase monotonically with collision energy, owing to the presence of energy barriers in both the entrance and exit channels. Despite being an exothermic reaction, the reaction probability remains low owing to the energy barrier. The OH vibrational excitation can promote the reaction activities as expected, because the O–H bond breaks in this reaction process. At the ground state, the ICSs do not show a significant increase with increasing collision energy, similar to the QCT results of Valero [57] and Garcia [58]. The ICSs obtained from Kudla and Schatz [39] were generally larger than our QCT results across the entire collision energy range. The vibrational mode *v*_CO_ = 1 remains unreactive and behaves as a spectator on the PES-OH + CO, different from Kudla and Schatz’s calculation. Our calculated ICSs at *v*_OH_ = 1, *v*_OH_ = 2, and *v*_OH_ = 4 are 5.6, 10.8, and 27.4 times that at the ground state, respectively. The reaction activity and the promotional effect become more pronounced with increasing vibrational energy level (*v*_OH_ = 1 < *v*_OH_ = 2 < *v*_OH_ = 4), since higher excited states provide more energy to facilitate the reaction process. Kohno et al. [46] found that the chemical reaction rate coefficients were increased with vibrational excitation, being enhanced by factors of 2.1, 3.8, 7.1, and 9.2 for the *v*_OH_ = 1, *v*_OH_ = 2, *v*_OH_ = 3, and *v*_OH_ = 4 modes, respectively, compared to that for *v*_0_. This deviation may be caused by the lack of the quantum effects in the QCT method. Both studies reveal that this reaction exhibits strong mode selectivity, where excitation of O–H bond significantly promotes the reaction, while the excitation of C–O bond acts as a spectator and does not enhance the reaction.

As shown in Table 1, both the ICSs of the OH + CO reaction and their ZPE-constrained ICSs generally increase with rising collision energy. At the ground state, the ICSs grow from 0.02 to 0.08 Å^2^ at *E*_c_ = 1 to20 kcal mol^−1^. The *v*_OH_ = 1 and *v*_O__H_ = 2 modes exhibit more pronounced growth, rising from 0.13 and 0.25 up to 0.41 and 0.86 Å^2^, respectively. The *v*_OH_ = 4 mode shows the most substantial increase, with ICS varying between 0.60 to 1.9 Å^2^. This indicates that higher vibrational excitation levels are more sensitive to collision energy. Under ZPE constraints, the ICS values for all modes remain largely consistent with their unconstrained counterparts. This suggests that ZPE effects have only a minor influence on the ICSs and do not alter the overall trend. Notably, the ICS of the *v*_CO_ = 1 mode is smaller than those of OH excited states across all collision energies, demonstrating that CO vibrational excitation is far less effective in promoting the reaction than in comparison with OH excitation. This finding further supports the dominant role of the OH stretching mode in driving the reaction, while CO essentially behaves as a spectator. The ZPE-constraint ICSs are similar to the original QCT ones, indicating that only a small portion of trajectories do not meet the ZPE-constraint; the ICSs are seldom affected.

**Figure 3:**
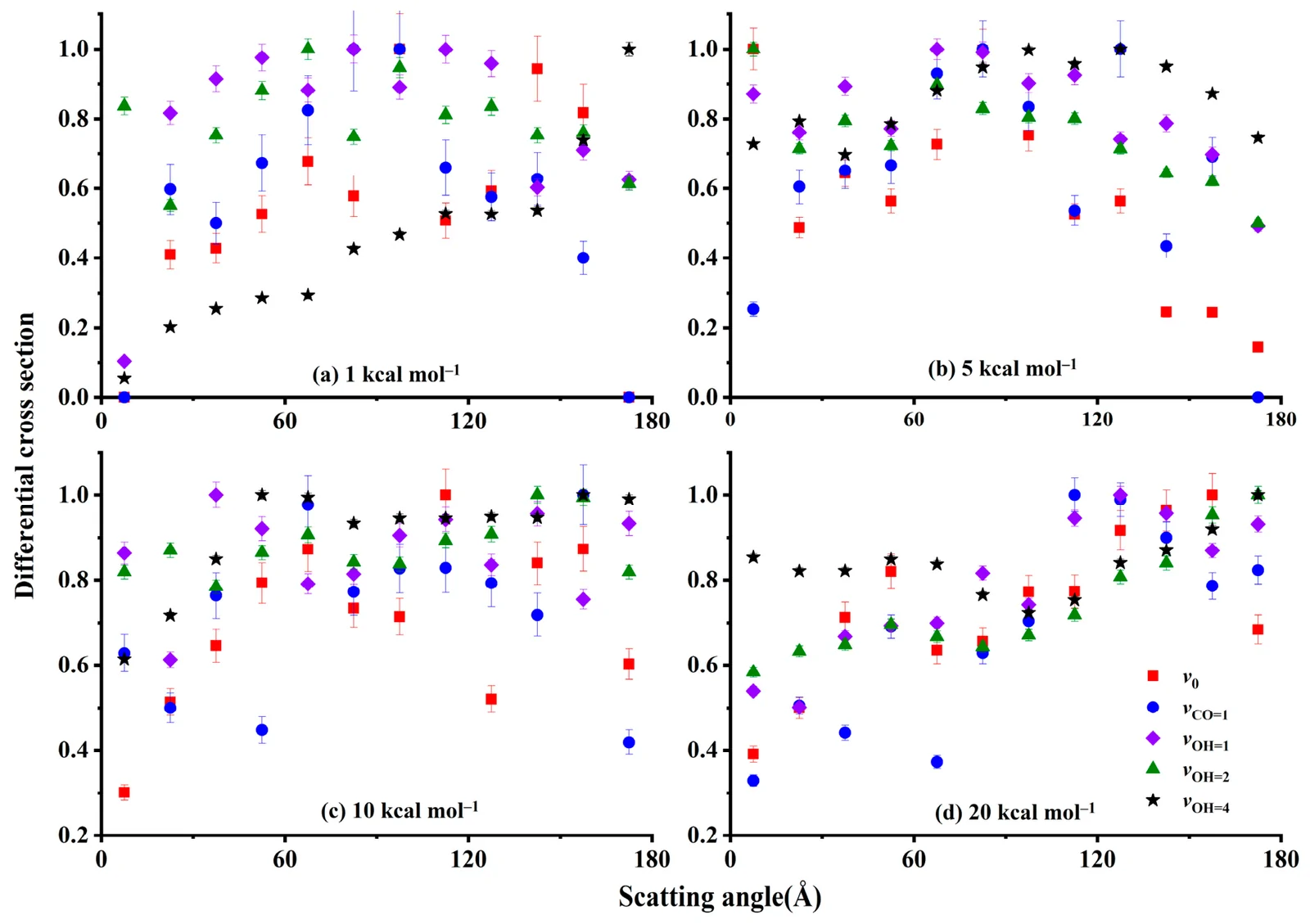
Relative DCSs of the OH + CO reaction for several different vibrational modes at different *E*_c_ (kcal mol^−1^).

Figure 3 presents the DCSs for the OH + CO → H + CO_2_ reaction as a function of the scattering angle, calculated at various initial vibrational states and collision energies. As shown in Figure 3a, side scattering dominates the angular distribution for the OH + CO reaction, and only a small number of events are found at the forward or backward scattering at *E*_c_ = 1 kcal mol^−1^. The DCS is dominated by backward scattering with considerable contributions from sideways direction (around 90°), and a small component from forward scattering (small angle) for *v*_OH_ = 4 mode. Small impact parameters result in large scattering angles, suggesting the rebound and direct mechanisms for the barrier-containing reaction. At *E*_c_ = 5 kcal mol^−1^, the scattering pattern is dominated by the forward direction, albeit with a certain degree of angular dispersion. At *E*_c_ = 10 kcal mol^−1^, the angular distributions tend toward isotropy for all vibrational modes. The experimental [36] and theoretical [58] observed an almost symmetric backward–forward double-peak structure with a slight preference for the forward and backward direction at *E*_c_ = 14.1 kcal mol^−1^, respectively. This suggests that the reaction is usually interpreted as a result of the formation of an intermediate complex whose lifetime is comparable to its rotational period. At *E*_c_ = 20 kcal mol^−1^, the angular distribution exhibits contributions across the full angular range, with a clear predominance of backward scattering. Meanwhile, the direct mechanism may dominate the reaction. In our previous work [14], the experimental measured and theoretical calculated DCSs showed scattering in both forward and backward directions, with a slight forward bias. Good, although not perfect, agreement between experiment and theory was reached. Specifically, the combined experimental-theoretical study found that a lower limit to the HOCO complex lifetime was calculated to be 0.5 and 0.8 ps for the higher and lower *E*_c_ experiments, respectively. The corresponding values obtained directly from the QCT calculations were 0.98 and 1.45 ps, respectively [14].

**Figure 4:**
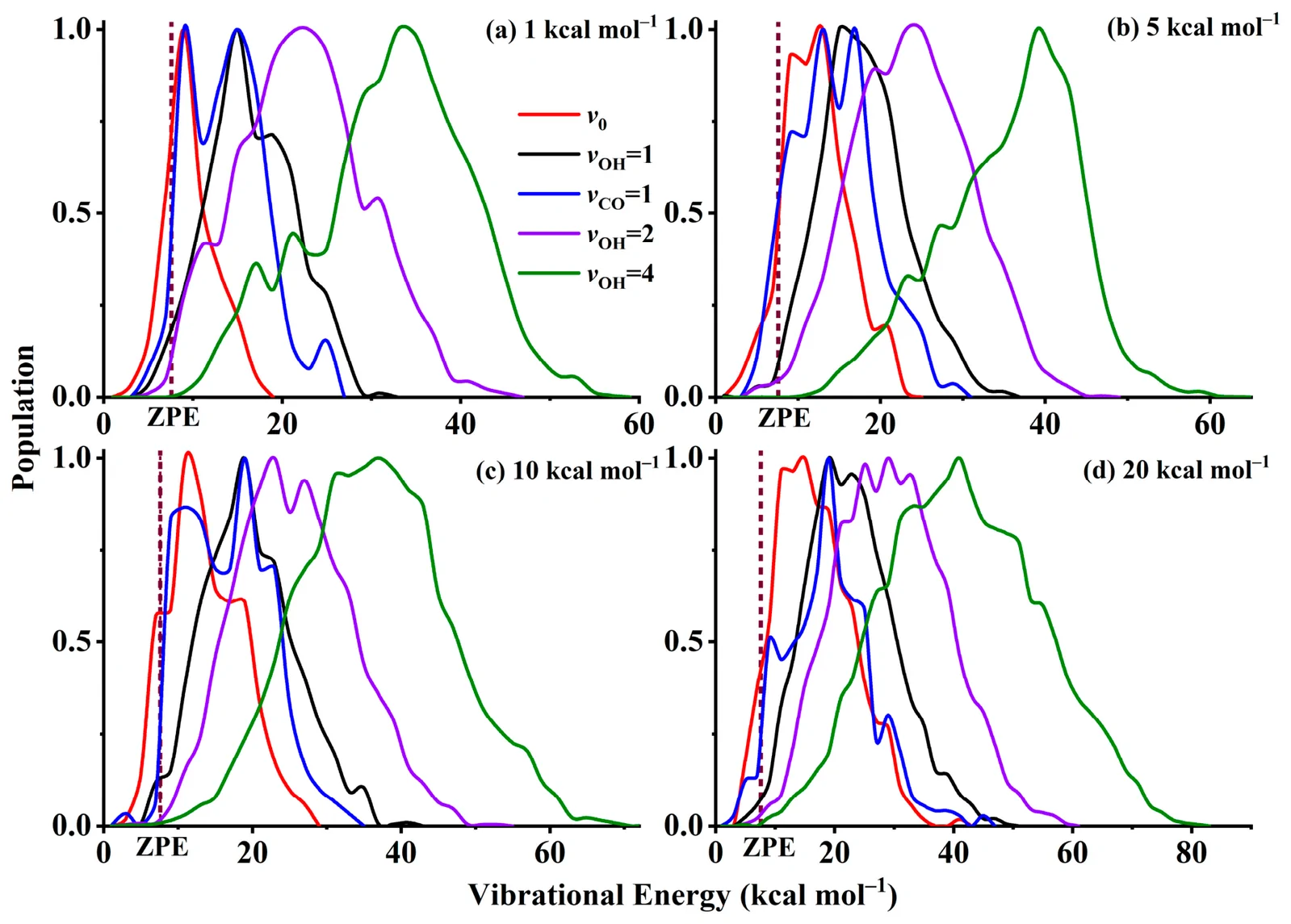
The product vibrational energy distributions of CO_2_ for the reaction OH + CO → H + CO_2_ at different vibration modes and collision energies.

Figure 4 illustrates the vibrational energy distributions of the CO_2_ product for the OH + CO reaction at *E*_c_ = 1, 5, 10, and 20 kcal mol^−1^, respectively. Each subplot compares distributions for the initial vibrational states: *v*_0_, *v*_OH_ = 1, *v*_CO_ = 1, *v*_OH_ = 2, and *v*_OH_ = 4. The vertical dashed lines marking the zero-point energy indicate that the results are consistent with the ZPE-constrained ICSs, and most of the vibrational energies of the products exceed the zero-point energy. The product vibrational energies distributions follows: *v*_0_ < *v*_CO_ = 1 < *v*_OH_ = 1 < *v*_OH_ = 2. The product vibrational energies increase significantly from *E*_c_ = 1 to 20 kcal mol^−1^. Not only the peak but also the shape is significantly shifted parallel to the high energy. The peak position of the highly vibrational excited state *v*_OH_ = 4 (green curve) lies distinctly higher than those of the other vibrational levels. This observation plainly demonstrates how collision energy and vibrational mode govern the product energy partitioning, and the energy partitioned into product vibrational motion.

**Figure 5:**
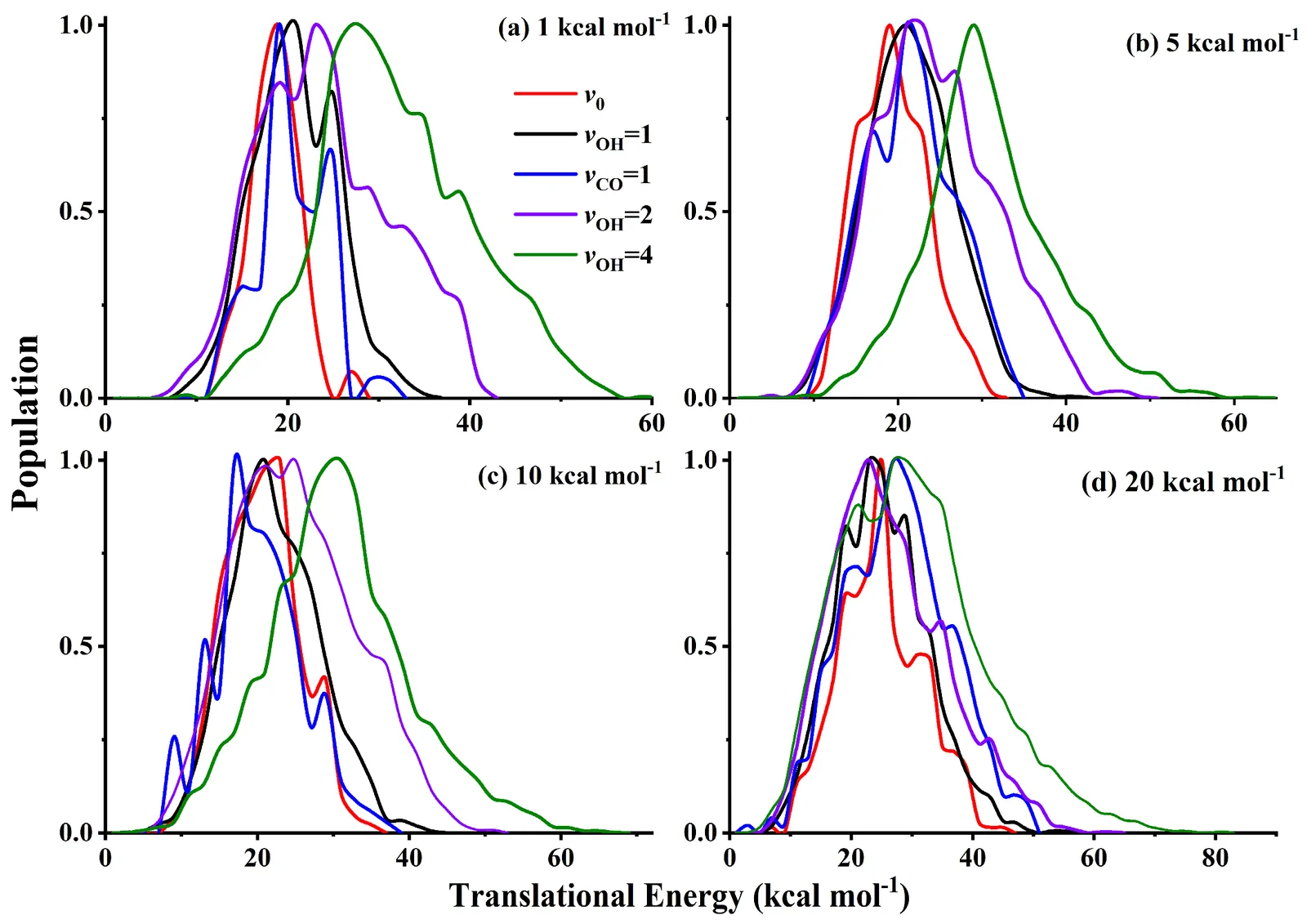
The product relative translational energy distributions of H and CO_2_ for the reaction OH + CO → H + CO_2_ at different vibrational modes and collision energies.

Figure 5 shows the relative translational energy distributions of the products for the OH + CO reaction at *E*_c_ = 1, 5, 10, and 20 kcal mol^−1^. Each subplot compares distributions for initial vibrational states *v*_0_, *v*_OH_ = 1, *v*_CO_ = 1, *v*_OH_ = 2, and *v*_OH_ = 4. Excitation of the OH vibrational mode shifts the translational energy distribution toward higher energy, and the *v*_OH_ = 4 curve peaks at the largest translational energy. The peak of the *v*_OH_ = 4 mode is located near 30 kcal mol^−1^, and its position remains largely unaffected by collision energy. As collision energy increases, all distributions become broadened and extend to higher translational energies, but the maximum distribution stays at around 20 kcal mol^−1^ for *v*_0_, *v*_OH_ = 1, *v*_CO_ = 1, and *v*_OH_ = 2 modes. This indicates that minor energy is partitioned into product relative translational motion. This is also consistent with the QCT values calculated on the YMS PES [58]. The calculated on the YMS PES had a maximum depending on the reactant collision energy (that was located at *E*_tr_ = 19 and 21 kcal mol^−1^ for reactant collision energies of 8.6 and 14.1 kcal mol^−1^, respectively). They also calculated that an increase in the CO rotational energy does not appreciably affect the product distributions [58].

### 2.2. OH + SO

**Figure 6:**
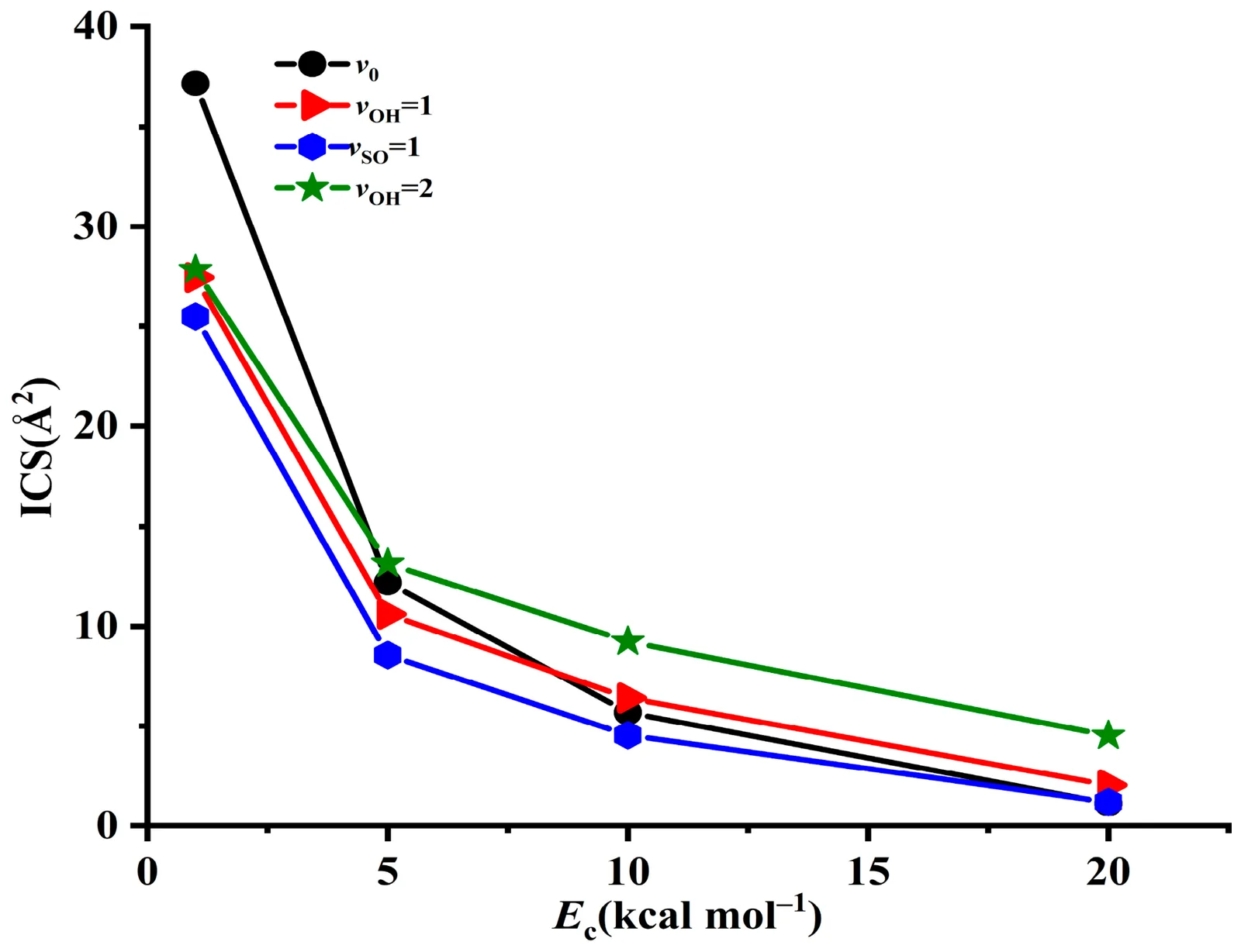
The integral cross-sections for the reaction OH + SO as a function of collision energies.

QCT calculations were carried out to analyze the variation in ICSs for the OH + SO reaction under different reactant vibrational modes, as depicted in Figure 6. The ICSs for all vibrational excitation modes decrease with increasing collision energy. This observation is consistent with an exothermic reaction mechanism lacking a barrier and aligns with the capture-trapping model. Accordingly, excitation of these three modes inhibits the reactivity. At *E*_c_ = 5 kcal mol^−1^, both *v*_OH_ = 1 and *v*_SO_ = 1 vibrational excitations tend to suppress the reaction. When collision energy rises to *E*_c_ = 10 kcal mol^−1^, only the *v*_SO_ = 1 mode produces slight inhibition. By contrast, *v*_OH_ = 2 mode exhibits a far stronger promotional impact than *v*_OH_ = 1 mode. It is speculated that the long-lived intermediate may facilitate intramolecular vibrational energy redistribution (IVR) under low collision energies. In contrast, at high collision energies, the lifetime of the complex could be greatly reduced, leaving insufficient time for energy relaxation. The vibrational energy pre-stored in the O–H bond may be directly channeled into bond-breaking. This offers one possible explanation for why the favorable vibrational excitation promotion appears exclusively within the high collision energy range.

Table 2 presents the ICSs of the radical reaction OH + SO under two computational schemes (without and with ZPE-constraint), at four collision energies and four vibrational excited states of reactants. At *E*_c_ = 1 kcal mol^−1^, the calculated ICS for the vibrational ground state is 37.17 Å^2^, indicating high reactivity. However, the ICSs with ZPE-constraint are 29.81 Å^2^, indicating that a portion of trajectories do not meet the ZPE-constraint; the ICSs are affected. The calculated ICS values for the other three vibrational excitation modes, *v*_OH_ = 1, *v*_SO_ = 1, and *v*_OH_ = 2, are 27.44, 25.49, and 27.83 Å^2^, respectively, smaller than that of the ground state. The corresponding ICSs with ZPE-constraint are 27.08, 24.73, 27.38 Å^2^ for _OH_ = 1, *v*_SO_ = 1, and *v*_OH_ = 2 modes. The ZPE influence is prominent at low energies and the *v*_0_ mode. After accounting for the zero-point energy constraint, the effective reaction energy barrier could be raised, leading to a slight reduction in integral cross-sections under most conditions.

**Figure 7:**
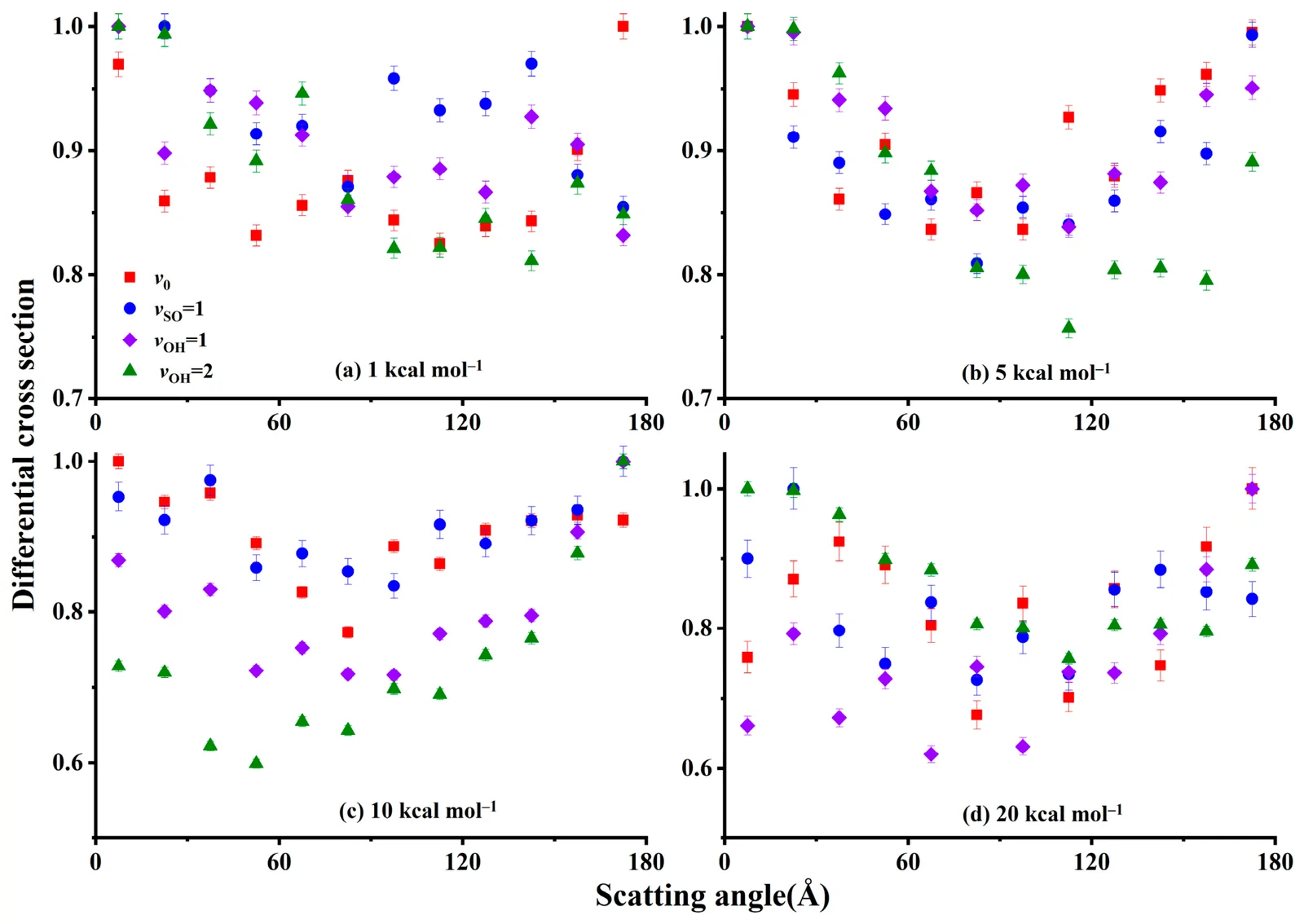
Relative DCSs of the OH + SO reaction for several different vibrational modes at different *E*_c_ (kcal mol^−1^).

Figure 7 presents the calculated DCSs for the OH + SO reaction at collision energies of 1, 5, 10, and 20 kcal mol^−1^, plotted as functions of the scattering angle for several vibrational modes. There are no experimental DCS and product-energy data for the OH + SO reaction to date. All DCSs are nearly isotropic distributions, suggesting a relatively long-lived complex that randomize the scattering directions. As collision energy increases, the DCSs gradually evolve toward prominent distinct double-peak structures with enhanced scattering intensity in both forward and backward directions. These DCSs differ from those observed in the OH + CO → H + CO_2_ reaction, which proceeds via HOCO and HCO_2_ intermediates. Such distinct scattering behaviors may be attributed to the topographical disparities in their PES between the two systems. In particular, the OH + SO reaction is essentially barrierless in both the entrance and exit channels, while OH + CO features nearly equivalent barriers for both. The HOSO intermediate well is substantially deeper than that of HOCO, with a well depth of 70.7 vs. 29.6 kcal mol^−1^.

**Figure 8:**
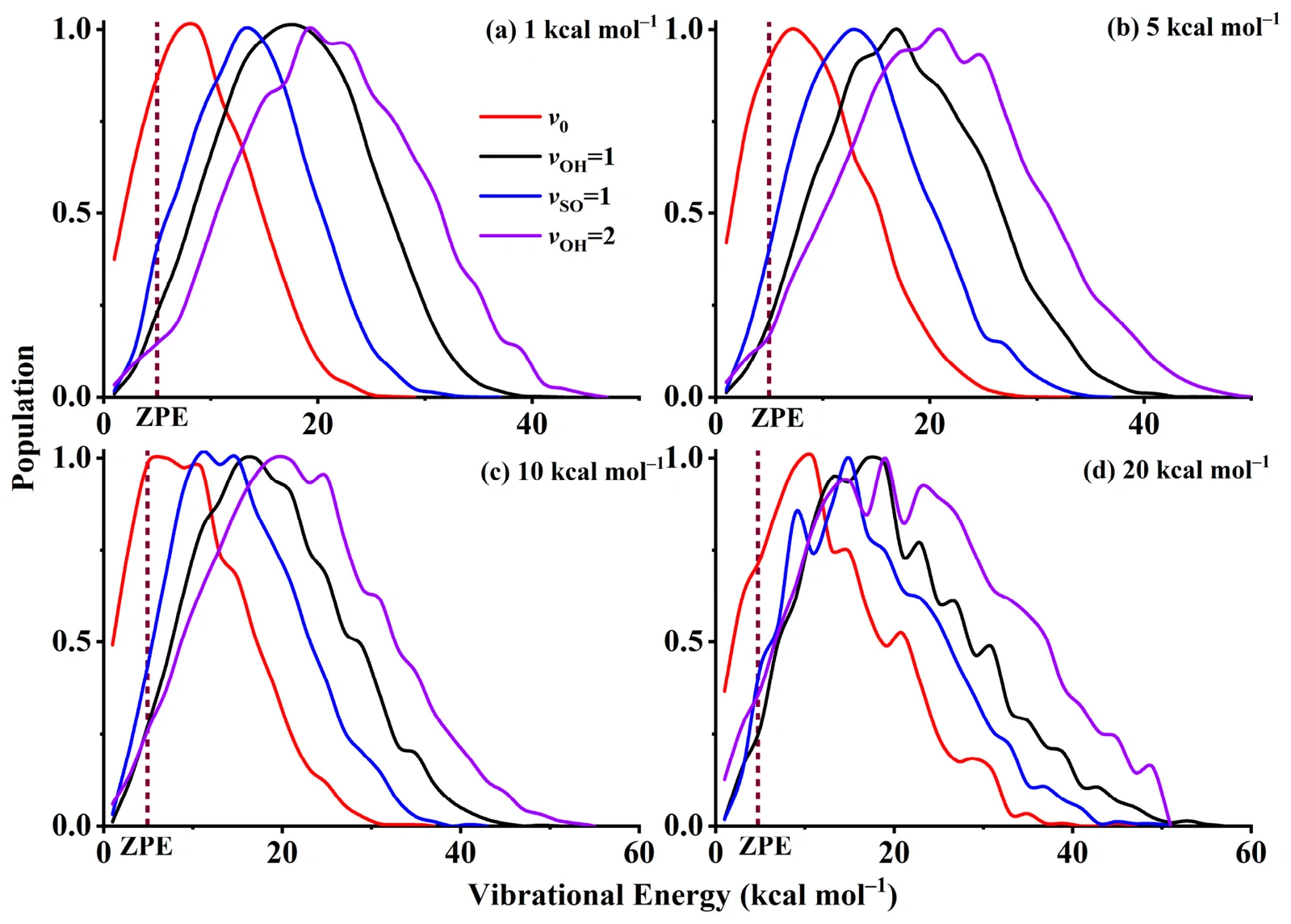
The product vibrational energy distributions of SO_2_ for the reaction OH + SO → H + SO_2_ at different vibration modes and collision energies.

The distributions of the SO_2_ product vibrational energy for the reaction OH + SO → H + SO_2_ are plotted in Figure 8a–d at *E*_c_ = 1, 5, 10 and 20 kcal mol^−1^. One can see that the product vibrational energy is increased significantly from *v*_0_ to *v*_OH_ = 2 among all energy range. Not only the peak but also the shape is significantly shifted parallel to the high-energy end. The ordering of the vibrational energies is as follows: *v*_0_ < *v*_CO_ = 1 < *v*_OH_ = 1 < *v*_OH_ = 2. The distributions of the product vibrational energies remain largely unaffected by collision energy. Furthermore, the ZPE-violating fraction in the distributions seem to be small, due to the exothermic nature of the reaction.

**Figure 9:**
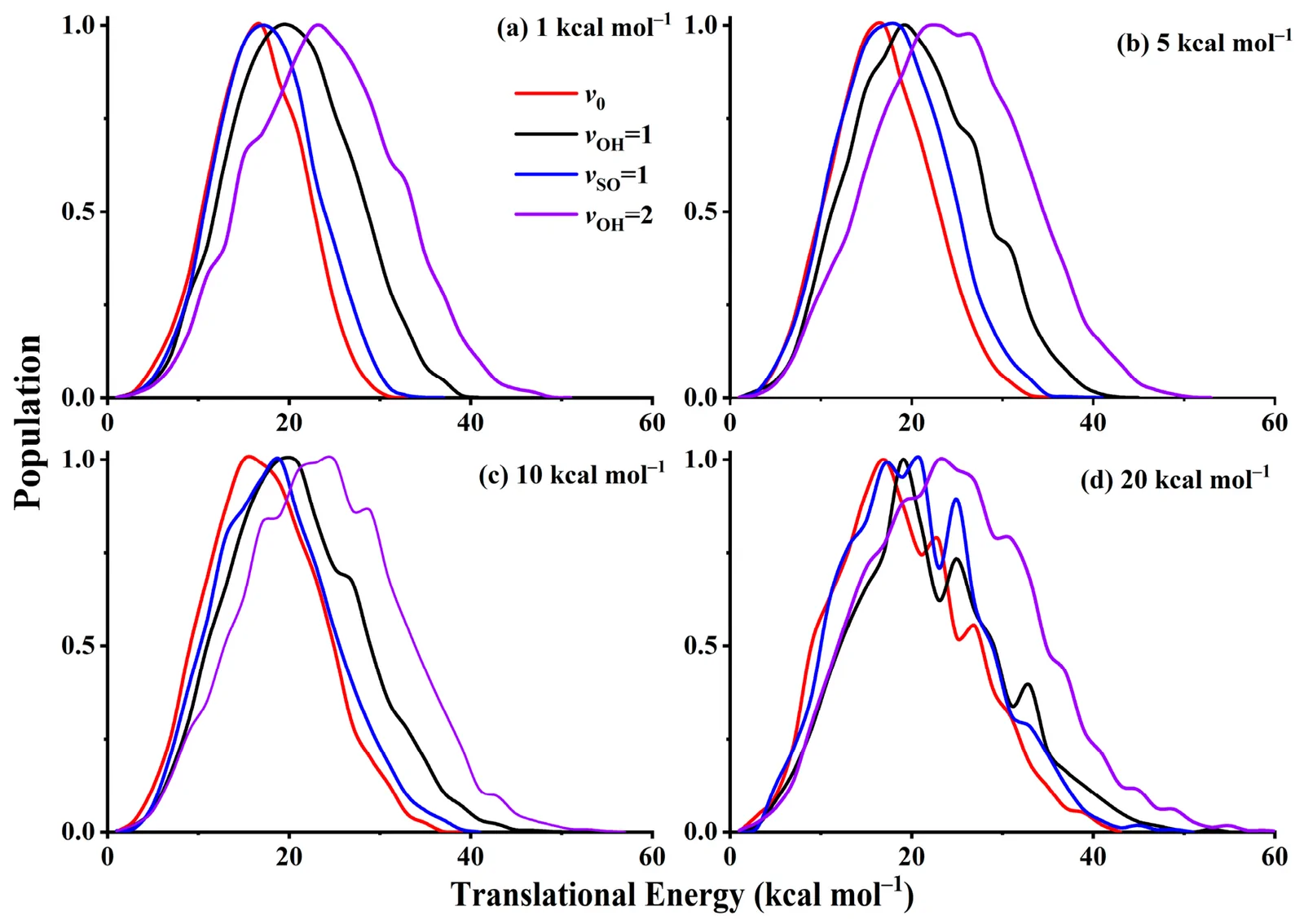
The product relative translational energy distributions of H and SO_2_ for the reaction OH + SO → H + SO_2_ at different vibrational modes and collision energies.

In contrast, the distributions of the SO_2_ product relative translational energies for the OH + SO → H + SO_2_ reaction are not affected much by the collision energy and vibrational level increased, as shown in Figure 9. Only the shapes of the distributions become a little wider with more collision energy available. The *v*_OH_ = 2 mode is slightly larger than those of the other vibrational modes.

**Figure 10:**
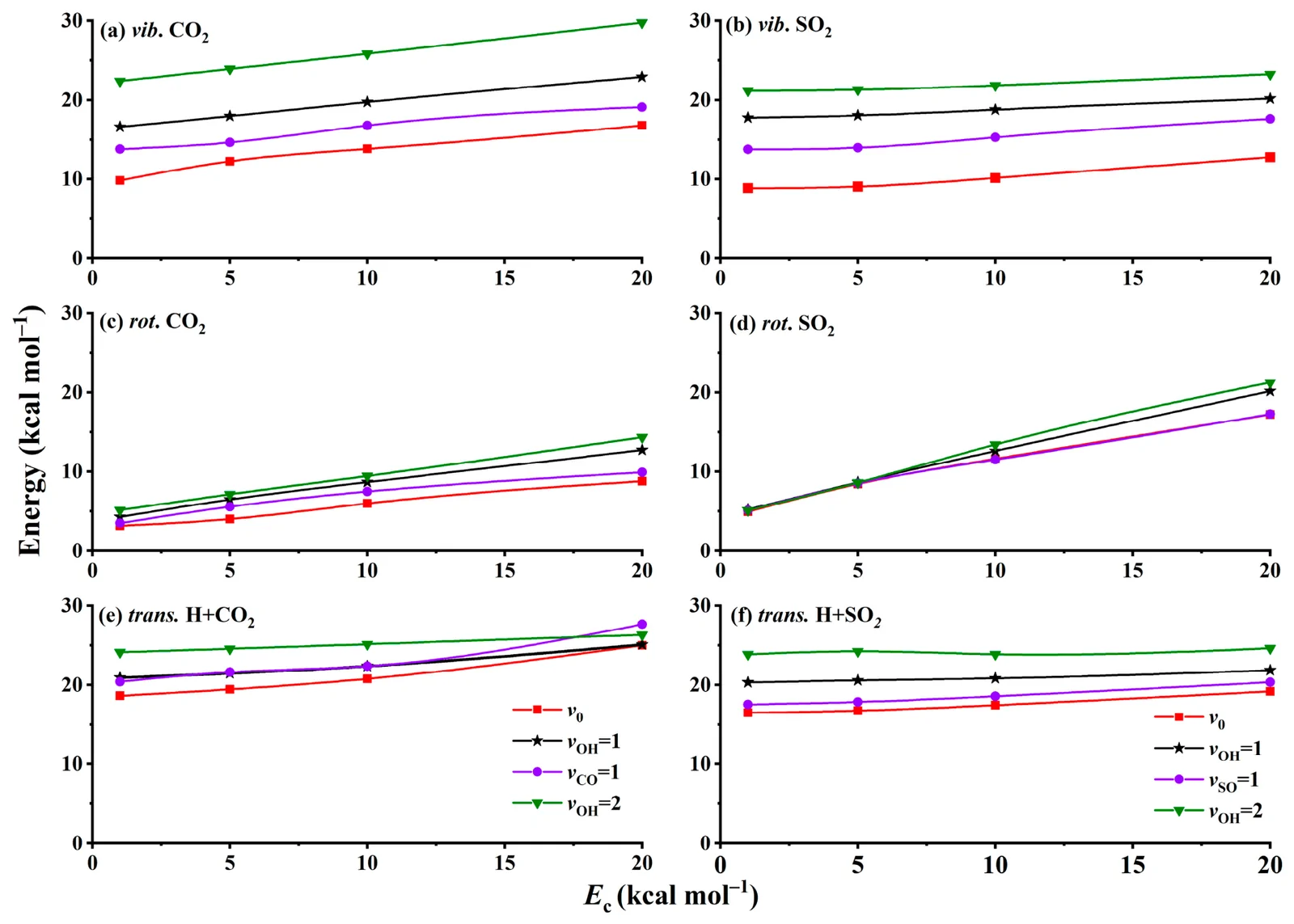
Average products’ rovibrational energies and the relative translational energies for CO_2_ and SO_2_.

The mode-specific product energy distributions of the CO_2_ and SO_2_ are summarized in Figure 10. As shown in Figure 10a,b, the average vibrational energies of both products grow with increasing collision energy. However, the vibrational energy growth gradient of CO_2_ is steeper than that of SO_2_. For both products, the ordering of average vibrational energies is as follows: *v*_0_ < *v*_CO_/*v*_SO_ = 1 < *v*_OH_ = 1 < *v*_OH_ = 2. Specifically, the average vibrational energy of CO_2_ ranges from 9.8 to 16.7 kcal mol^−1^ at ground states, while for SO_2_ ranges from 8.8 to 12.8 kcal mol^−1^ as *E*_c_ increases from = 1 to 20 kcal mol^−1^. The average rotational energies of the two products also increase with collision energy, yet the rotational energies of CO_2_ rise more slowly than those of SO_2,_ as shown in Figure 10c,d. The average rotational energy of CO_2_ depends on the mode of vibrational excitation, with a general order as follows: *v*_0_ < *v*_CO_ = 1 < *v*_OH_ = 1 < *v*_OH_ = 2. However, the average rotational energies for SO_2_ remain nearly consistent across all vibrational excited modes at *E*_c_ = 1 and 5 kcal mol^−1^. At *E*_c_ ≥ 10 kcal mol^−1^, the energy ordering becomes *v*_0_ ≈ *v*_SO_ = 1 < *v*_OH_ = 1 < *v*_OH_ = 2. For the *v*_0_ mode, the average rotational energy of CO_2_ ranges from 3.15 to 8.79 kcal mol^−1^, while the average rotational energy of SO_2_ ranges from 4.93 to 17.20 kcal mol^−1^, as *E*_c_ increases from 1 to 20 kcal mol^−1^. The relative translational energies of the two products do not vary significantly with collision energy, as shown in Figure 10e,f. The relative translational energies of the product H + SO_2_ follow the order *v*_0_ < *v*_SO_ = 1 < *v*_OH_ = 1 < *v*_OH_ = 2. For the product H + CO_2_, the *v*_OH_ = 2 mode has the maximum translational energy at 1, 5, and 10 kcal mol^−1^. At *E*_c_ = 20 kcal mol^−1^, the *v*_co_ = 1 mode takes the leading position. Similarly, the *v*_0_ mode always corresponds to the minimum energy case and matches the value of the *v*_OH_ = 1 mode at *E*_c_ = 20 kcal mol^−1^. A striking contrast in product energy disposal can be identified between the two reaction systems. The energy of CO_2_ is predominantly partitioned into vibrational degrees of freedom, whereas SO_2_ is mainly stored in rotational modes. This fundamental difference may be traced to the disparate dynamical behaviors of the reaction intermediates on their respective potential energy surfaces. It is plausible that the long-lived HOSO and/or HSO_2_ complexes (much deeper than HOCO or HCO_2_) could provide sufficient time for nearly complete intramolecular vibrational energy randomization, allowing efficient energy flow into the rotational manifold of the departing SO_2_ fragment. In contrast, the shorter-lived HOCO intermediate might limit the extent of vibrational-to-rotational energy transfer, which offers one possible explanation for the observed vibrational dominance in CO_2_ products.

## 3. Computational Methods

Quasi-classical trajectory (QCT) calculations were performed based on the PES-OH + CO and PES-OH + SO [16,19] interfaced to the VENUS [59,60] chemical dynamics program. Initial collision energies were chosen to be *E*_c_ = 1, 5, 10, and 20 kcal mol^−1^ at the ground state (*v*_0_) and vibrational levels of OH (*v*_OH_ = 1~4), CO (*v*_CO_ = 1), and SO (*v*_SO_ = 1).

The initial rotational quantum numbers of the reactants were set to zero, and the initial relative orientations of the reactants were randomly sampled. The initial separation distance between the reactants was 10.0 Å. Trajectories were terminated when the products or reactants reached a distance of 10.5 Å. These initial and termination criteria are sufficiently large to account for long-range interactions between the reactants and products for the aforementioned initial conditions. Trajectories were integrated using a combination of the fourth-order Runge–Kutta and sixth-order Adams–Moulton algorithms. The time step was chosen to be 0.05 fs, and an energy convergence criterion during propagation was set to 10^−3^ kcal mol^−1^. For each specified initial condition, the maximum impact parameter *b*_max_ was determined from 10^4^ test trajectories. The impact parameter *b* was sampled as *b* = *b*_max_ξ^0.5^, where ξ is a uniformly distributed random number in the interval (0, 1). For the OH + CO and OH + SO reactions, 10^5^ and 5 × 10^4^ trajectories were run for each collision energy and vibrational mode to ensure reasonably small statistical errors. The reaction probability *P*_r_ (*E*_c_) = *N*_r_/*N*_total_, which is the ratio of the number of reactive trajectories to the total number of trajectories. The reactive integral cross-section at the collision energy *E*_c_ was determined according to the following formula:

(1) $$\sigma_{r}(E_{c})=\pi b_{\max}^{2}(E_{c})P_{r}\left(E_{c}\right)$$

The statistical error is estimated by

(2) $$\Delta =\sqrt{(N_{total}-N_{r})/N_{total}N_{r}}$$

Table 3 lists the statistical errors of different vibrational modes at *E*_c_ = 1, 5, 10, and 20 kcal mol^−1^. For the OH + CO reaction, only a small number of the reactive trajectories are generated at low collision energies. At *E*_c_ = 1 kcal mol^−1^, the statistical errors for *v*_0_ and *v_C_*_O_ = 1 modes are 0.10 and 0.12, respectively, slightly larger than other modes. Due to the high reactivity of the OH + SO reaction, all statistical errors are less than 0.03.

The differential cross-section is determined according to

(3) $$\frac{d\sigma_{r}}{d\Omega }=\frac{\sigma_{r}P_{r}(\theta )}{2\pi \sin(\theta )}$$

The scattering angle *θ* is defined as

(4) $$\theta =\cos^{-1}\left(\frac{{\vec{\nu }}_{i}\cdot {\vec{\nu }}_{f}}{\left|{\vec{\nu }}_{i}\right|\left|{\vec{\nu }}_{f}\right|}\right)$$

Here, $\vec{v}$ denotes the relative velocity vector with the subscripts ‘*i*’ and ‘*f*’ for ‘initial’ and ‘final’, ${\vec{v}}_{i}={\vec{v}}_{\mathrm{OH}}-{\vec{v}}_{\mathrm{CO}}$, ${\vec{v}}_{f}={\vec{v}}_{H}-{\vec{v}}_{{\mathrm{CO}}_{2}}$ for OH + CO reaction, ${\vec{v}}_{i}={\vec{v}}_{\mathrm{OH}}-{\vec{v}}_{\mathrm{SO}}$, ${\vec{v}}_{f}={\vec{v}}_{H}-{\vec{v}}_{{\mathrm{SO}}_{2}}$ for OH + SO reaction. In this way, *θ* = 0° and *θ* = 180° correspond to the forward and backward scattering, respectively. *P_r_* (*θ*) is the normalized probability for the scattering products at the angle *θ*. For each initial condition with a specified collision energy, DCS (*θ*) is calculated by using a bin size of 15°.

Table 4 lists the *b*_max_ values as a function of collision energy and vibrational mode. Across all vibrational modes, the *b*_max_ values decrease with increasing collision energy. For the OH + CO system, except for the *v_C_*_O_ = 1 mode at *E*_c_ = 10 kcal mol^−1^, the *b*_max_ values for all excited states are greater than or equal to those of the ground states at the same collision energy. For the OH + SO system, the ground state yields the largest *b*_max_ (6.7 Å) at *E*_c_ = 1 kcal mol^−1^, while the excited states exhibit slightly smaller values. The *b*_max_ values for *v*_0_, *v*_OH_ = 1, *v*_SO_ = 1, and *v*_OH_ = 2 modes at *E*_c_ = 1 kcal mol^−1^ and 20 kcal mol^−1^ are 6.7, 6.5, 6.6, 6.6 Å and 3.6, 3.6, 3.4, 3.6 Å, respectively. The long-range attractive interactions between OH and SO facilitate mutual orientation and enhance reactivity, which could make the indirect capture mechanism dominant in the reaction at low collision energies.

Due to the intrinsic drawback of the QCT approach, the vibrational energies of the products may be lower than their zero-point energies (ZPEs). The zero-point energies and vibrational quantum state energies were determined according to the following formula:

(5) $$E=(v+\frac{1}{2})hf$$

where *f* is the harmonic vibrational frequency, and v=0 is the vibrational quantum state, which can take values of 0, 1, 2, …, and v=0, $E=\frac{1}{2}hf$, which is called the zero-point vibrational energy. Therefore, the ZPEs for OH, CO, SO, CO_2_, and SO_2_ are 5.56, 3.21, 1.73, 7.60, and 4.39 kcal mol^−1^.

Many methods [61,62,63] have been developed to correct the ZPE leakage issue in QCT. The so-called soft ZPE constraint method [64,65,66] was employed in this work. Only trajectories yielding products with vibrational energies no less than the sum of their ZPEs were considered. For the OH + CO → H + CO_2_ reaction, we use the trajectories whose vibrational energy of the CO_2_ is larger than 7.60 kcal mol^−1^ in the analysis. For the OH + SO → H + SO_2_ reaction, we use the trajectories whose vibrational energy of the SO_2_ is larger than 4.39 kcal mol^−1^. The reactive probability is quite low, and only a small fraction of reactive trajectories yield products with vibrational energy below the ZPE. As an illustration, we take the *v*_CO_ = 1 mode at *E*_c_ = 1 kcal mol^−1^ (the case with the lowest reactivity) as an example; only 3 among 72 reactive trajectories produce products with vibrational energy below their ZPE. We deleted those reactive trajectories that do not meet the soft ZPE criterion. Hence, the soft-ZPE constraint for the reactive trajectories does not affect the initial Boltzmann distribution of the reactants.

**Figure 11:**
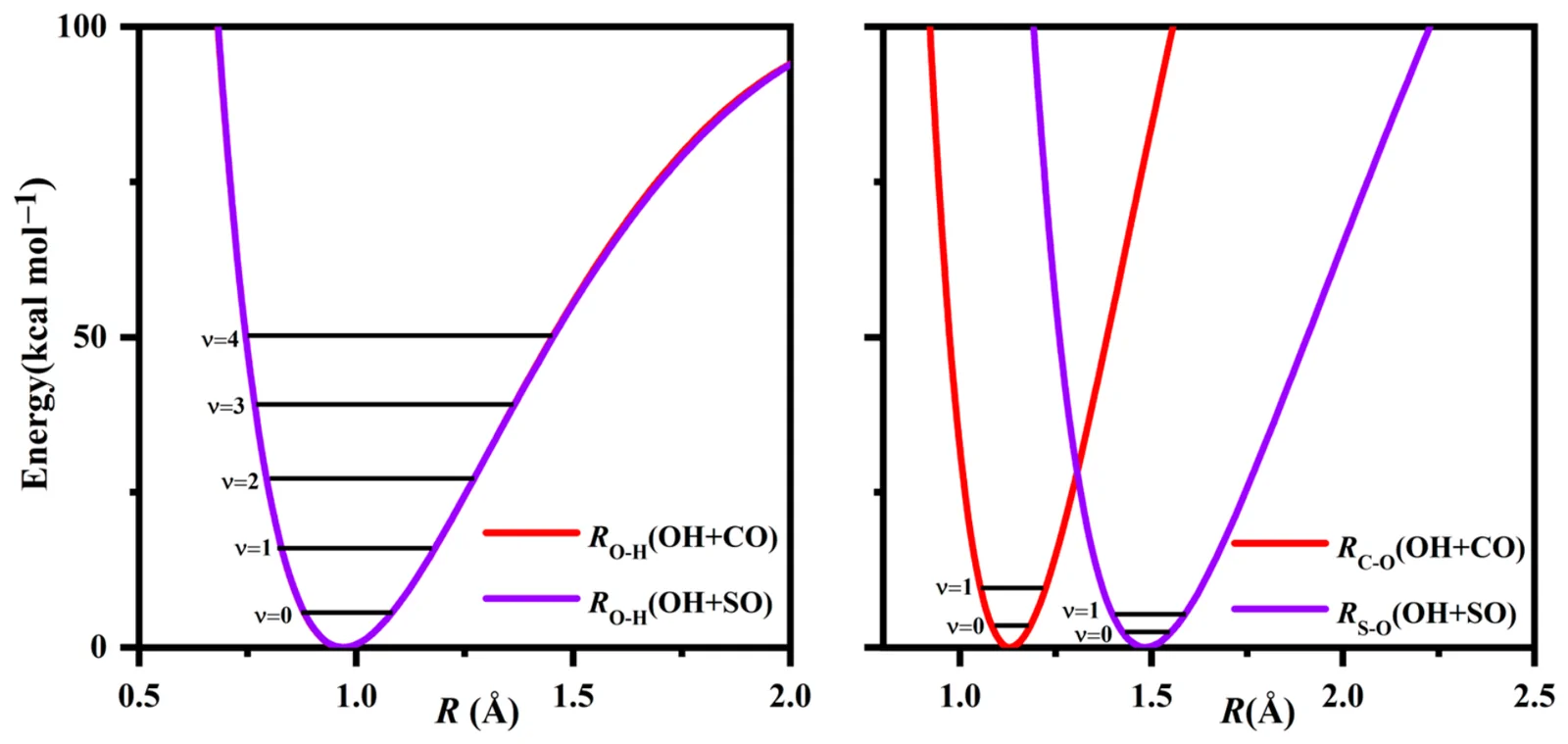
The stretching coordinates of the *R*_OH_ (**a**), *R*_CO,_ and *R*_SO_ (**b**), with the reactants separated by 12 Å and other coordinates fixed at the HOSO and *cis*-HOCO equilibrium.

Figure 11 displays the stretching coordinates of the: *R*_OH_, *R*_SO,_ and *R*_C__O_. The intermolecular distance between OH and XO (X = C/S) is fixed at 12 Å and other coordinates constrained at the HOSO and cis-HOCO equilibrium geometries. The potential energy and corresponding bond length of each vibrational energy level *v* (*v*_OH_ = 1 ~ 4, *v*_SO/CO_ = 0,1) are displayed explicitly. As illustrated in Figure 11a, the potential energy curves of *R*_OH_ for the OH + CO (red) and OH + SO (purple) reaction systems are identical. The two potential-well minima are located near 0.97 Å, consistent with the equilibrium bond-length of the OH radical. The zero-point vibrational frequency of the OH radical is 3743 cm^−1^, which is equivalent to 5.56 kcal mol^−1^. The stored vibrational excitation energy can reach 50.36 kcal mol^−1^ at mode *v*_OH_ = 4. Figure 11b reveals obvious discrepancies in the equilibrium positions of the respective potential wells. The well minimum for *R*_CO_ (red, OH + CO) is located at 1.13 Å, whereas the minimum for *R*_SO_ (purple, OH + SO) lies at 1.48 Å. The zero-point vibrational energies of CO and SO are both lower than that of OH. Their potential energies exhibit strong reliance on bond-length displacement, and this sensitivity is particularly pronounced for the C−O bond.

## 4. Conclusions

In this work, quasi-classical trajectory calculations have been performed on two full-dimensional PESs. A systematic comparative analysis of mode-specific reactivity and product energy disposal is presented for two related oxygen-transfer reactions: OH + CO → H + CO_2_ and OH + SO → H + SO_2_. The effects of their PES topology, collision energy, and reactant vibrational excitation on reactivity and product energy disposal are investigated.

The ICSs of the two reactions exhibit opposing collision energy dependence, accompanied by distinct mode-specific effects. For the OH + CO reaction, the ICSs increase monotonically with rising collision energy. Excitation of the O–H stretching modes effectively promote the reaction, with higher vibrational levels yielding progressively stronger enhancement: *v*_OH_ = 4 > *v*_OH_ = 2 > *v*_OH_ = 1. In contrast, the C–O stretching mode behaves as a spectator; its excitation barely alters reaction activity. For the barrierless reaction OH + SO, the ICSs decrease monotonically as collision energies increase. Excitation of the O–H stretching mode (*v*_OH_ = 2) promotes reactivity at moderate-to-high collision energies. The two systems also display profoundly distinct scattering behaviors in their DCSs. The OH + CO reaction system shows mixed direct/indirect dynamical mechanisms, characterized by symmetric double peaks DCS profiles, with backward scattering becoming dominant at high collision energies. The OH + SO reaction system might result from its deep potential well and the long-lived complex yields nearly isotropic DCS distributions at low collision energies, which gradually evolve into forward–backward double-peak structures at higher collision energies. A similarly marked divergence is also observed in the product energy disposal dynamics. For the OH + CO reaction, the majority of the available excess energy is preferentially deposited into the vibrational degrees of freedom of the CO_2_ product. In contrast, most of the excess available energy is preferentially allocated to the robrational energy of the SO_2_ product for the OH + SO reaction. This could be attributed to the extensive intramolecular energy redistribution enabled by the long-lived intermediate complexes. Initial vibrational excitation further enhances the internal energy of the corresponding products, with the most pronounced effect observed for O–H stretching modes. The results indicate that differences in PES barriers, well depths, and associated complex-forming dynamics play a major role in determining the contrasting reaction and energy-disposal behaviors. The present work represents an exploration of these distinct dynamical behaviors, enabled by two state-of-the-art, highly accurate ab initio PESs.

## Figures and Tables

**Table 1:** The ICSs (Å^2^) and ZPE-constraint ICSs (Å^2^) for the OH + CO reaction at different vibrational modes and different *E*_c_ (kcal mol^−1^).

	OH + CO					OH + CO (ZPE-Constraint)				
*E*_c_ *Mode*	*v*_0_	*v*_OH_ = 1	*v*_CO_ = 1	*v*_OH_ = 2	*v*_OH_ = 4	*v*_0_	*v*_OH_ = 1	*v*_CO_ = 1	*v*_OH_ = 2	*v*_OH_ = 4
1	0.02	0.13	0.02	0.25	0.60	0.02	0.13	0.02	0.24	0.60
5	0.05	0.28	0.04	0.52	1.46	0.04	0.28	0.03	0.52	1.46
10	0.06	0.31	0.04	0.62	1.64	0.06	0.31	0.04	0.62	1.64
20	0.08	0.41	0.10	0.86	1.93	0.08	0.40	0.10	0.86	1.93

**Table 2:** The ICSs (Å^2^) and ZPE-constraint ICSs (Å^2^) for the OH + SO reaction at different vibrational modes and *E*_c_ (kcal mol^−1^).

	OH + SO				OH + SO (ZPE-Constraint)			
*E*_c_ *Mode*	*v*_0_	*v*_OH_ = 1	*v*_SO_ = 1	*v*_OH_ = 2	*v*_0_	*v*_OH_ = 1	*v*_SO_ = 1	*v*_OH_ = 2
1	37.17	27.44	25.49	27.83	29.81	27.08	24.73	27.38
5	12.18	10.61	8.55	13.14	9.62	10.46	8.27	12.92
10	5.69	6.41	4.54	9.24	4.58	6.31	4.39	9.04
20	1.12	2.04	1.19	4.52	0.97	1.99	1.16	4.37

**Table 3:** The statistical errors of different vibrational modes at *E*_c_ = 1, 5, 10, and 20 kcal mol^−1^.

	OH + CO					OH + SO			
*E*_c_ *Mode*	*v*_0_	*v*_OH_ = 1	*v*_C__O_ = 1	*v*_OH_ = 2	*v*_OH_ = 4	*v*_0_	*v*_OH_ = 1	*v*_SO_ = 1	*v*_OH_ = 2
1	0.10	0.04	0.12	0.03	0.02	0.01	0.01	0.01	0.01
5	0.06	0.03	0.08	0.02	0.01	0.01	0.01	0.01	0.01
10	0.06	0.03	0.07	0.02	0.01	0.01	0.01	0.02	0.01
20	0.05	0.02	0.04	0.02	0.01	0.03	0.02	0.03	0.01

**Table 4:** The *b*_max_ values (Å) of vibrational modes at *E*_c_ = 1, 5, 10, and 20 kcal mol^−1^.

	OH + CO					OH + SO			
*E*_c_ *Mode*	*v*_0_	*v*_OH_ = 1	*v*_CO_ = 1	*v*_OH_ = 2	*v*_OH_ = 4	*v*_0_	*v*_OH_ = 1	*v*_SO_ = 1	*v*_OH_ = 2
1	2.7	2.9	2.8	3.0	2.9	6.7	6.5	6.6	6.6
5	2.4	2.6	2.6	2.6	2.8	4.2	4.2	4.2	4.3
10	2.6	2.6	2.5	2.7	2.7	3.9	3.9	3.9	3.9
20	2.4	2.6	2.5	2.6	2.7	3.6	3.6	3.4	3.6

## Data Availability

All data generated or analyzed during this study are included in this published article.
